# Animal models and conserved processes

**DOI:** 10.1186/1742-4682-9-40

**Published:** 2012-09-10

**Authors:** Ray Greek, Mark J Rice

**Affiliations:** 1 Americans For Medical Advancement (www.AFMA-curedisease.org), 2251 Refugio Rd, Goleta, CA, 93117, USA; 2 Department of Anesthesiology, University of Florida College of Medicine, PO Box 100254, Gainesville, FL, 32610-0254, USA

**Keywords:** Anesthesia, Animal models, Cancer, Complexity, Conserved processes, Systems biology

## Abstract

**Background:**

The concept of conserved processes presents unique opportunities for using nonhuman animal models in biomedical research. However, the concept must be examined in the context that humans and nonhuman animals are evolved, complex, adaptive systems. Given that nonhuman animals are examples of living systems that are *differently complex* from humans, what does the existence of a conserved gene or process imply for inter-species extrapolation?

**Methods:**

We surveyed the literature including philosophy of science, biological complexity, conserved processes, evolutionary biology, comparative medicine, anti-neoplastic agents, inhalational anesthetics, and drug development journals in order to determine the value of nonhuman animal models when studying conserved processes.

**Results:**

Evolution through natural selection has employed components and processes both to produce the same outcomes among species but also to generate different functions and traits. Many genes and processes are conserved, but new combinations of these processes or different regulation of the genes involved in these processes have resulted in unique organisms. Further, there is a hierarchy of organization in complex living systems. At some levels, the components are simple systems that can be analyzed by mathematics or the physical sciences, while at other levels the system cannot be fully analyzed by reducing it to a physical system. The study of complex living systems must alternate between focusing on the parts and examining the intact whole organism while taking into account the connections between the two. Systems biology aims for this holism. We examined the actions of inhalational anesthetic agents and anti-neoplastic agents in order to address what the characteristics of complex living systems imply for inter-species extrapolation of traits and responses related to conserved processes.

**Conclusion:**

We conclude that even the presence of conserved processes is insufficient for inter-species extrapolation when the trait or response being studied is located at higher levels of organization, is in a different module, or is influenced by other modules. However, when the examination of the conserved process occurs at the same level of organization or in the same module, and hence is subject to study solely by reductionism, then extrapolation is possible.

## Background

Marc Kirschner and John Gerhart introduced the concept of facilitated variation and conserved core processes in their book, *The Plausibility of Life*[1], in order to explain how novelty arises in evolution. Motivated by advances in evolutionary and developmental biology (evo devo), these investigators proposed that conserved processes are ubiquitous in eukaryotes but pointed out that by using conserved processes differently, for example by differently regulating the genes that code for the processes, expressing the genes differently, varying the sequences or combination of genes or transcription factors, novelty can arise. Mutations in the genes that regulate the conserved processes can accomplish this novelty. Moreover, by adjusting the regulatory genes, the organism can evolve with fewer mutations than would be the case if a trait had to arise *de novo* or from mutations in structural genes. This has implications for using nonhuman animals (hereafter referred to simply as animals) as models for humans in biomedical research. One should expect to discover information regarding conserved processes in humans by studying animal models. We sought to determine whether limits exist on this method and if so what those limits are.

## Methods

We surveyed the relevant literature including philosophy of science, biological complexity, conserved processes, evolutionary biology, comparative medicine, anti-neoplastic agents, inhalational anesthetics, and drug development journals in order to determine the appropriate role for animal models when studying conserved processes. Philosophy of science is relevant to our discussion as it includes the premises and assumptions on which research is then based. A study or method can be *methodologically* sound but if the premises are incorrect, then the study loses much if not all of its value. The drug development literature was searched because the final application of much research is targeted intervention via drugs hence that literature can inform regarding the success of a practice or modality. The literature concerning biological complexity and conserved processes was surveyed as it directly relates to the issue being explored. All of this must be placed into the context of evolutionary biology in order to better explain the findings. We chose inhalational anesthetics and anti-neoplastic agents as examples because of the well-known conserved nature of these agents.

## Results

### Animal models

The use of models has a long history in science, which led philosopher of science Richard Braithwaite to warn that: “The price of employment of models is eternal vigilance” [2]. In this section, we will explore what animal models are, how they can be used in scientific investigation, including biomedical research, and discuss classification schemes. In this article, we will address the use of predictive animal models in light of the concepts of complex systems, personalized medicine and pharmacogenomics, and evolutionary biology. We will then explore what this implies when using animal models to study conserved processes.

Models are important for scientific pursuits and can take the form of abstract models, computational models, heuristic models, mathematical models, physical models such as scale models, iconic models, and idealized models. Models can also be divided on the basis of whether they are used to replicate a portion of the item being modeled or are used to test hypotheses or interpret aspects of a theory. Examples of historically important models include Watson and Crick’s physical model of DNA, Pauling’s model of chemical bonds, Bohr’s solar system model of the atom, and the billiard ball model of gases. More recent models include the computer model of the brain, mathematical models of disease spread, and Lorenz’s model of the atmosphere.

Robert Hinde observed that models:

● Should be different from the thing being modeled, because if it is not, the modeler might assume that all properties demonstrated by the model exist in the thing being modeled;

● Are usually less complicated than the thing being modeled;

● Are more readily available than the thing being modeled, and;

● “pose questions, suggest relations, or can be manipulated in ways not possible with the original” [3].

In light of the importance of models, some philosophers of science assert that the study of models *per se* has been neglected by the philosophy of science community. Frigg and Hartmann [4] state: “What fills in the blank in ‘*M* represents *T* if and only if ____,’ where *M* is a model and *T* a target system?” Moreover, how one classifies models and what criteria must be fulfilled in order for *M* to be considered a specific type of model has arguably not been adequately addressed by the philosophy of science community. Yet another problem with the *philosophy of models* is the relationship between theory and model [4]. We maintain that this lack of scholarly attention to models has played a role in what we see as the confusion surrounding the use of animals as models.

Animal models are physical models and can be further classified based on various features and uses. For example, they can be distinguished by the phylogenetic distance of the model species from humans. Animal models can also be classified based on fidelity—how well the model resembles humans—as well as based on validity—how well what you think you are measuring corresponds to what you really are measuring. Animal models can also be considered based on reliability—the precision and accuracy of the measurement [5]. Hau explains that animal models can be categorized as spontaneous, induced, transgenic, negative and orphan. Hau states: “The majority of laboratory animal models are developed and used to study the cause, nature, and cure of human disorders” [[6] p3]. This is important as Hau further states that animal models can be used to predict human responses: “A third important group of animal models is employed as *predictive* models. These models are used with the aim of discovering and quantifying the impact of a treatment, whether this is to cure a disease or to assess toxicity of a chemical compound. The appropriateness of any laboratory animal model will eventually be judged by its capacity to explain and predict the observed effects in the target species” [6]. Others agree that predicting human response is a common use for animal models [7-12]. For example, Heywood stated: “Animal studies fall into two main categories: predictive evaluations of new compounds and their incorporation into schemes designed to help lessen or clarify a recognised hazard” [13].

Animals are utilized for numerous scientific purposes (see ]Table 1) and one of the authors (Greek) has addressed these various uses in previous publications [14-20]. One cannot have a meaningful discussion regarding the utility of animal models unless one specifies the category under discussion. For example, areas in which animal models have been successfully employed include the evaluation of a phenomenon that can be described by the physicochemical properties of the organism, the study of basic physiologic functions, and the study of other traits that can be described by the use of conversion factors based on the body surface area of the organism. In general, animal models can be successfully employed in categories 3–9 in Table 1. However, animal models have failed to be predictive modalities for human response to drugs and disease [13-16,18,21-41], depicted by categories 1 and 2 in Table 1. (The authors have addressed this failure in numerous publications and, because an exploration for this failure is not the purpose of the article, we refer the reader to those publications [14-20,23] even though we realize that some view this position as controversial [7,11,42-44].) This is not to say that a species can never be found in retrospect that mimics an outcome in humans. Such a species usually can be identified, however retrospective correlation is obviously not the same as prediction [45-47]. Moreover, any process or modality claiming to be predictive can be evaluated by use of the binomial classification table and equations in Table 2 (as illustrated in Table 3[48]). Such calculations are commonly used in science [49-53].

**Table 1:** **Categories of animal use in science and research**[16]

	
1.	As predictive models for human disease
2.	As predictive models to evaluate human exposure safety in the context of pharmacology and toxicology (e.g., in drug testing)
3.	As sources of ‘spare parts’ (e.g., aortic valve replacements for humans)
4.	As bioreactors (e.g., as factories for the production of insulin, or monoclonal antibodies, or the fruits of genetic engineering)
5.	As sources of tissue in order to study basic physiological principles
6.	For dissection and study in education and medical training
7.	As heuristic devices to prompt new biological/biomedical hypotheses
8.	For the benefit of other nonhuman animals
9.	For the pursuit of scientific knowledge in and of itself

**Table 2:** Binary classification test

		**Gold standard**	
		**GS+**	**GS-**
**Test**	**T+**	**TP**	**FP**
	**T-**	**FN**	**TN**
Sensitivity = TP/(TP + FN)			
Specificity = TN/(FP + TN)			
Positive Predictive Value = TP/(TP + FP)			
Negative Predictive Value = TN/(FN + TN)			
T- = Test negative			
T + = Test positive			
FP = False positive			
TP = True positive			
FN = False negative			
TN = True negative			
GS- = Gold standard negative			
GS + = Gold standard positive			

The binary classification test allows calculations for determining how well a test or practice compares with reality or the gold standard.

**Table 3:** Example of binary classification values

		**Gold standard (human)**	
		**GS+**	**GS-**
**Test**	**T+**	**22**	**26**
	**T-**	**22**	**30**
Sensitivity = 22/(22 + 22) = 0.5			
Specificity = 30/(26 + 30) = 0.54			
Positive Predictive Value = 22/(22 + 26) = 0.46			
Negative Predictive Value = 30/(22 + 30) = 0.58			

Binary classification values for cardiovascular toxicity test in monkeys from 25 compounds also tested in humans [48]. Note the values are approximately what would be expected from a coin toss.

When judging the predictive value of a modality, one is not using the term *predict* in the same sense as when describing how hypotheses generate predictions to be tested. The predictive value of a commonly used modality usually is known, or can be ascertained, for example the positive and negative predictive value of x-ray computed tomography (commonly referred to as a CT scan) for diagnosing pneumothorax (a rupture of, or interference in, the pleural membrane which allows air to enter the pleural space and thus interferes with breathing) approaches 1.0 (is accurate for diagnosing the condition in 100% of cases).

Animal models as used in biomedical research, can also be categorized as causal analogical models (CAMs) or as heuristic or hypothetical analogical models (HAMs) [54-59]. The use of animal models to predict human response to drugs and disease, in accordance with categories 1 and 2 in Table 1, would be an example of using animals as CAMs. Analogical models in general include the hydraulic model of economies and the computer model of the brain and can be further divided based on various criteria [4]. Causalism or causal determinism dates to Aristotle who stated: “what is called Wisdom is concerned with the primary causes and principles.” Causalism can be summarized succinctly, as “everything has a cause.” This notion of causation was the basis for animal models as can be appreciated by the writings of Claude Bernard [60], considered the father of animal modeling since the 19^th^ century. Bernard’s thoughts on animal models are an extension of Aristotle via the determinism of Descartes and Newton [61]. Causal determinism and the principle of uniformity led to the concept, still accepted by many animal modelers today, that the same cause would result in the same effect in qualitatively similar systems. This line of thinking was in keeping with the creationist thinking of 19^th^ century French physiologists, including Bernard, who rejected Darwin’s Theory of Evolution [60,62,63]. The notion of causal determinism and the principle of uniformity combined with the rejection of evolution led to the belief in the interchangeability of parts. Therefore, if one ascertained the function of the pancreas in a dog, he could directly extrapolate that knowledge to the function of the pancreas in humans, once scaling for size had been factored in [14,63,64]. Unfortunately, this linear thinking persists as the baboon heart transplant to Baby Fae illustrates. The operation was performed by the creationist surgeon Leonard Bailey of Loma Linda University in 1984 [[65] p162-3].

We acknowledge that the concept of *causation* is problematic [66]. Russell suggested it be abandoned in 1913 [67] and it is clearly more useful for linear systems than complex systems. While an exhaustive explanation and discussion of the controversies surrounding causation would occupy more space than is available for this article (see Bunge [61] for such an analysis) we should note that a more current explanation for causation is that of a “first order approximation.” Causation is usually discussed in the context of a *chain* of causes. Bunge summarizes current thinking: “neodeterminism . . . asserts in this connection that causation is only one among several interrelated categories concurring in real processes” [61]. This principle is appreciated even more fully in complex systems. Current thinking notwithstanding, the use of animal models assumes the Cartesian concept of causation in that a causal model assumes a deterministic causal relationship between variables. We will explore this thinking and show that even in the traditional context there are problems with using animal models to discover “causal” relationships. These problems are increased exponentially when placed in the context of complex systems.

Based on the writings of LaFollette and Shanks [[58]p63], we suggest the following in order for a model to be considered a CAM. *X* (the model) and *Y* (the subject being modeled) share properties {*a…e*}. In X, these properties are associated with, and thought relevant to, state *S1*. *S1* has not been observed directly in *Y*, but *Y* likely also has would exhibit *S1* under the same conditions as X. This concept is illustrated in Table 4. LaFollette and Shanks [58] state that, “there should be no causally-relevant disanalogies between the model and the thing being modeled.” Unfortunately, causally relevant disanalogies do exist among species and even within a species, which leads to different states or outcomes, as illustrated in Table 4. We again paraphrase LaFollette and Shanks [[58] p112] and suggest that two more conditions must be met for a model to qualify as a CAM: the shared properties {*a,…,e*} must have a causal relationship with state *S1* and be the only causally relevant properties associated with *S1*. As Table 4 illustrates, the commonalities between the humans and chimpanzees are insufficient to qualify chimpanzees as CAMs for human response to HIV infection. (For more on animal models of HIV/AIDS see [14,68].) As we will show, animals and humans are evolved complex systems and as such exhibit the properties of robustness and redundancy; hence numerous “causes” can result in the same effect and the same perturbation can result in different outcomes. Because of this and other properties of complex systems, we should expect different species to exhibit different causal relationships.

**Table 4:** Causal analogical models

**X, the model**	**Y, the system being modeled**	**Shared properties between X and Y**	**Perturbation to the model**	**Outcome in model**	**Outcome in system being modeled**
Animal system (for example, *Pan troglodytes*)	Human system				
		a. Genes. >90% of nucleotide sequences identical.	Exposure to HIV.	State *S1*. Mild illness of limited duration.	AIDS. State *S1* is *not* shared despite the presence of shared, relevant properties.
		b. Immune system. Many commonalities. Constructed on generally the same plan.			
		c. White blood cells present and function similarly.			
		d. Receptors on white blood cells also present and function similarly.			
		e. Shared intracellular components of white blood cells.			

Shared properties *a . . . e* for humans and chimpanzee do not result in state *S1* also being shared.

Correspondingly, Giere, Bickle, and Mauldin [69] note that some question the use of causal models in the study of humans because humans are complex systems whereas casual models assume a deterministic system: an outcome in a simple system is fixed by the variables. The problems of determining causation are further explored by Bunge [61] in his neodeterminism explanation alluded to above and his analysis is highly relevant to this discussion. While we will attempt to contrast the traditional deterministic view of causality in light of complexity science, this article will not do justice the current thinking on causation and we refer the reader to Bunge [61] for a fuller explanation.

Giere, Bickle, and Mauldin suggest a probabilistic relationship instead of a 100% causal relationship for the model: “C is a positive causal factor (probabilistic) for E in an individual, I, characterized by residual state, S, if in I the probability of E given C is greater than the probability of E given Not-C.” Likewise, LaFollette and Shanks raise the question as to whether animal models can be *weak CAMs*: “Begin with two systems S_1_ and S_2_. S_1_ has causal mechanisms {a,b,c,d,e}, S_2_ has mechanisms {a,b,c,x,y}. When we stimulate sub-system {a,b,c} of S_1_ with stimuli s_f_ response r_f_ regularly occurs. We can therefore infer that were we to stimulate sub-systems {a,b,c}of S_2_ with s_f_ r_f_ would probably occur” [[58] p141]. LaFollette and Shanks then explain that this outcome will be highly probable if and only if {a,b,c} are causally independent of {d,e} and {x,y}.

Again we anticipate problems in using animal models as weak CAMs, even in the traditional deterministic-causation view, because, as we shall discuss, various properties of complex systems will likely give rise to difficulties in isolating subsystems, which would be required for an animal model to be a weak CAM. These problems have been referred to as *causal/functional asymmetry* and mandates caution in extrapolating data between species. Kirschner and Gerhart give an example of this:

"The case of the octopus and the human camera eye has been looked into, and the lessons are clear. Underneath the gross anatomical similarities are many differences. The eye derives from different tissues by different developmental means. Although both structures use the same pigment (rhodopsin) for photoreception, and both send electrical signals to the brain, we now know that the intervening circuitry is completely different [[1] p240-01]."

Independent evolution has also produced spindle neurons in species as diverse as humans and cetaceans. Spindle neurons connect parts of the brain involved in higher cognition and were thought to only occur in primates but have recently been discovered in cetaceans, such as humpback whales and fin whales, as well as elephants [70-72]. Convergent evolution, the acquisition of the same trait in different lineages, is also important when considering the role of animal models.

### Evolved complex systems

Reductionism is a method of study that seeks to break a system down into its component parts, study each part individually, and then reach a conclusion about the system as a whole or at least the role of the individual part. Descartes introduced the concept and it has proven effective for ascertaining many facts about the material universe. Conversely, the clockwork universe of Descartes has not held up to scrutiny on all levels. Quantum mechanics, relativity, chaos, and complexity have revealed the stochastic nature of the supposedly clockwork, deterministic universe. Regrettably, while physicists recognized the limitations of reductionism, biologists were uncritically embracing it. Francis Crick extended reductionism to all aspects of biology when he stated: “The ultimate aim of the modern movement in biology is to explain all biology in terms of physics and chemistry” [73]. Biological reductionism arguably reached its zenith in the Human Genome Project (HGP) [74,75] and, ironically, the consequences of the HGP—that humans have a relatively small number of genes—have, in large part, been responsible for a re-examination of the role of reductionism in biology. This has been especially true for human pathophysiology where animals are used as models for humans.

Systems can be categorized as simple or complex. The world of Newton and Descartes was largely confined to simple systems hence reductionism functioned well for discovery. At some levels, the components of a complex system can be simple systems and thus are subject to study by reductionism while at other levels these simple systems combine to make complex systems thus necessitating study of the intact whole. Mazzocchi points out that when reductionism takes a component out of its natural environment it has consequences for extrapolating the results back to the organism as a whole: “But this extrapolation is at best debatable and at worst misleading or even hazardous. The failure of many promising drug candidates in clinical research shows that it is not always possible to transfer results from mice or even primates to humans” [76].

While evolution is defined as a change in allele frequency over time, complexity science can be defined as “the study of the behaviour of large collections of simple, interacting units, endowed with the potential to evolve with time” [77,78]. Living organisms are complex systems that have highly variable evolutionary histories and as such are best modeled using nonlinear differential equations. The difficulty with this approach is that the values for many of the factors are unknown; hence solving the equation is impossible [49,77].

Animals and humans are examples of living complex adaptive systems and as such exhibit the following properties [79-97]:

1. Complex systems are composed of many components. Some of these components may be simple systems, but many are complex systems. These components exist on many scales and interact extensively with each other. A complex system is a “system of systems.”

2. The components can be grouped as modules. For example, the following could be considered as modules: the cell; the various processes in a cell; gene networks; gene-gene interactions; gene-protein interactions; protein-protein interactions; organs; and all the factors that influence the natural history of a disease. However, failure in one module does not necessarily spread demise to the system as a whole as redundancy and robustness (see #s 5 and 6 below) also exist and the various modules also communicate with each other.

3. The different components of a complex system are linked to and affect one another in a synergistic manner. There is positive and negative feedback in a complex system [93].

4. A complex system demonstrates hierarchal levels of organization [98,99]. These levels range from the subatomic to the molecular to the whole individual to collections of individuals [100]. Emergence (see # 13 below) occurs at each level; therefore, even a complete understanding of the lower level is insufficient for explaining the upper level. The various levels interact such that there is both upward causation and downward causation. In order to understand a particular level, one must alternate between looking at the components and looking at the whole while taking into account the connections between each [76,101]. Moreover, the various levels may respond differently to the same perturbation.

The various levels of organization are important when considering which responses to specific perturbations can be extrapolated among species. Living complex systems have numerous properties that can be studied without consideration of the fact that the whole, intact organism is a complex system. Some systems or components follow only the laws of physics, or even simple geometry, while others are best described by their physicochemical properties or just by chemistry. Some properties of complex systems can be described simply by math formulas. Growth, for example, can be described as geometrical in some cases and exponential in others. The surface area of a body increases by the square of the linear dimensions while the volume increases by the cube. This is a consequence of geometry and is important in physiology, in part, because heat loss is proportional to surface area while heat production is proportional to volume. Haldane stated: “Comparative anatomy is largely the story of the struggle to increase surface in proportion to volumes” [102]. For example, chewing increases the surface area of food, the rate the small bowel absorbs nutrients and other chemicals depends in part on the surface area of the small bowel, and air sacs in the lungs rely on surface area for gas exchange, as do capillaries.

Allometry is the study of the relationship of body size to shape. Examples of allometric laws include Kleiber’s law: *q*_0_ ~ *M*^¾^ where *q*_0_ is metabolic rate and is proportional to *M,* body mass, raised to the ¾ power. The rate *t,* of breathing and heart contractions are proportional to *M*, body mass, raised to ¼ power: *t* ~ *M*^¼^. Further, many physiological functions affect or depend on surface area.

Levels of organization can also be described based on whether they are primarily chemical reactions and hence subject to analysis by chemistry. Reactions or perturbations that involve the denaturation of proteins should affect all systems, be they simple or complex, similarly because at this level of organization other factors do not come into play. Exactly what effects sulfuric acid would have in a person over an extended period of time are irrelevant as it denatures protein more or less immediately. Perhaps species differences would manifest if small amounts of H_2_SO_4_ were infused over long periods of time, but the immediate effects are the same across species because of the chemical properties of the acid.

Animals can be successfully used for numerous purposes in science (see 3–9 in Table 1). One of the purposes for which animals can be successfully used is to evaluate phenomena that can be described by the physicochemical properties of the organism. The same applies to basic physiologic functions. There are physiological parameters that can be applied across species lines by the use of conversion factors based on the weight or surface area of the organism. There are also properties of organisms that can be anticipated by the physical or chemical properties of the substance acting on the organism. All of these are instances of successfully treating a complex system as if it were a simple system. Problems arise however, as the level of organization under study increases. Allometric scaling based on body surface area (BSA), for example, does not include differences that manifest at higher levels of organization for example in the elimination or metabolism of drugs. Different levels of organization can be acted on by single factors or many factors but perturbations of simple systems, or systems that can be described as simple on the level or organization being affected, should produce similar results.

5. Complex systems are robust, meaning they have the capacity to resist change. This can be illustrated by the fact that knocking out a gene in one strain of mouse may produce negligible effects while being lethal to another strain. Gene pleiotropy is an additional example [103].

6. Complex systems exhibit redundancy. For example, living systems exhibit redundancy of some genes and proteins [103].

7. Complex systems are dynamic. They communicate with, and are acted on by, their environment.

8. Complex systems exhibit self-organization, which allows adaptation to the environment [85,104-106]. The intact cell is a prime example of this property.

9. Complex systems are dependent on initial conditions. The well-known example of the butterfly flapping its wings and causing a weather catastrophe on the other side of the earth—the butterfly effect—is an example of dependence on initial conditions. An example in living complex systems would be that very small differences in genetic makeup between two systems could result in dramatic differences in response to the same perturbation. For example, monozygotic twins raised in the same environment may have different predispositions to diseases such as multiple sclerosis and schizophrenia [107-110]. Additionally, the above-mentioned observation that knocking out a gene results in different outcomes in two stains of mice illustrates the concept that small differences in initial conditions—genetic makeup—can mean the difference between life and death [93,103,111,112].

10. The initial conditions of a complex living system are determined, in part, by evolution. Various species have different evolutionary histories and thus are differently organized complex systems. Initial conditions can be different, despite the exact same genes, secondary to modifier genes, differences in regulation or expression of genes, epigenetics, and mutations among others factors. For example, small epigenetic changes probably account for the dissimilarities between monozygotic twins in terms of disease susceptibility [107,108,113-116].

11. Perturbations to complex systems result in effects that are nonlinear [99]. Large disturbances may result in no change to the system while minor perturbations may cause havoc [76,105]. Efforts to describe complex systems in terms of linear cause and effect relationships are prone to failure [117]. Extrapolating *among* complex systems is even more problematic because of nonlinearity, along with the other factors described.

12. The whole of a complex system is greater than the sum of the parts; hence, some processes and or perturbations are not amenable to study by reductionism.

13. Complex systems have emergent properties that cannot be predicted even in light of full knowledge of the component parts.

Animal models have historically been utilized for the prediction of human responses to drugs and disease and this use has also been the justification for animal use in research in general [118,119]. But because various levels of organization and different modules can be acted on by the same perturbation, in order to evaluate whether an animal model can be used as a predictive modality, one needs to understand the levels affected by the perturbation, what rules are being followed at those levels, and whether the system is simple or complex at the respective levels. Empirical evidence, explained and placed in context by theory developed from complexity science and evolutionary biology, suggests animal models cannot predict human responses to drugs and disease [14-16,18,57,58,120], despite the presence of shared physicochemical properties and conserved processes.

### Conserved processes

Theodosius Dobzhansky famously stated: “Nothing in biology makes sense except in the light of evolution.” We want to examine the consequences that various characteristics of evolved complex systems, such as modules and different levels of organization, have on processes conserved by evolution in terms of determining the response of whole organisms to perturbations. Conserved processes and genes are the subject of much interest today [1,121-133]. Kirschner and Gerhardt state: “all organisms are a mixture of conserved and nonconserved processes (said otherwise, or changing and unchanging processes)” [[1] p34-35]. Conserved *processes* are not reactions to the laws of physics or the determination of properties of an organism as they relate to chemistry or geometry. Nevertheless, conservation reaches across phyla and even kingdoms. Kirschner and Gerhardt have pointed out that processes conserved include those involved in cell function and organization, development, and metabolism and that these processes are similar in animals, yeast, and bacteria. They note that novelty has been the result of using the conserved processes in different ways rather than inventing completely new processes [[1] p34-35]. This has critical implications for what can be learned from interspecies study.

Housekeeping genes in general perform the same function; make the same proteins, in mice, frogs or humans. The role of FOX transcription factors is conserved among species [134] as is the role of Sarco(endo)plasmic reticulum (SER) Ca^2+^ ATPases (SERCA) pumps [135]. Modules have also been conserved. The fin module of the modern fish for example, arose roughly 400 million years ago and has been conserved ever since [[1] p65].

Conserved processes include core genes like those in the homeobox that are involved in the same developmental processes. Because these processes and genes are conserved among species, we could reasonably expect the same outcome from the same perturbation, regardless of the species containing these processes. But is this the case? In 1978 Lewis [136] published his seminal work on the anterior-posterior layout of *Drosophila*. This was followed in 1984 by the discovery of the homeobox by McGinnis et al. [137]. The field of evo devo developed in large part from this work. In the last decade, enormous strides have been made as a result of research in evo devo and the various genome projects. The results of such research have revealed an enormous genetic similarity among mammals. At the level of the genes centrally involved in development, e.g., the homeobox genes, bilaterians are virtually identical. The homeobox class of genes [138] are conserved across species lines, functioning in early cellular organization and anterior-posterior body plan layout [139]. There are important differences however. For example, there are nine Hox genes in flies but thirty-nine in mammals. Pertinently, we understand how modifications (gene duplications, deletions, changes in the regulatory processes and so forth) to these conserved processes have resulted in the evolution of different body types and indeed different species [138,140-142].

MicroRNA (miRNA) has been found in essentially all species from *Caenorhabditis elegans* to humans and plays a large role in gene regulation [143-145]. Apparently, over 50% of miRNAs are conserved across species lines in vertebrates [145]. An important consideration for drug development, however, is the fact that even though miRNA is conserved, up to 50% of miRNAs differs among vertebrates. This is important when considering the use of animals as predictive human models. Furthermore, miRNA expression levels change when tissues deteriorate from a healthy state to a diseased state [146-152]. Thus the exact role of miRNA may differ intra-individually depending on age and disease. Hence, we see both inter-species and intra-individual differences with respect to this conserved process.

**Figure 1:**
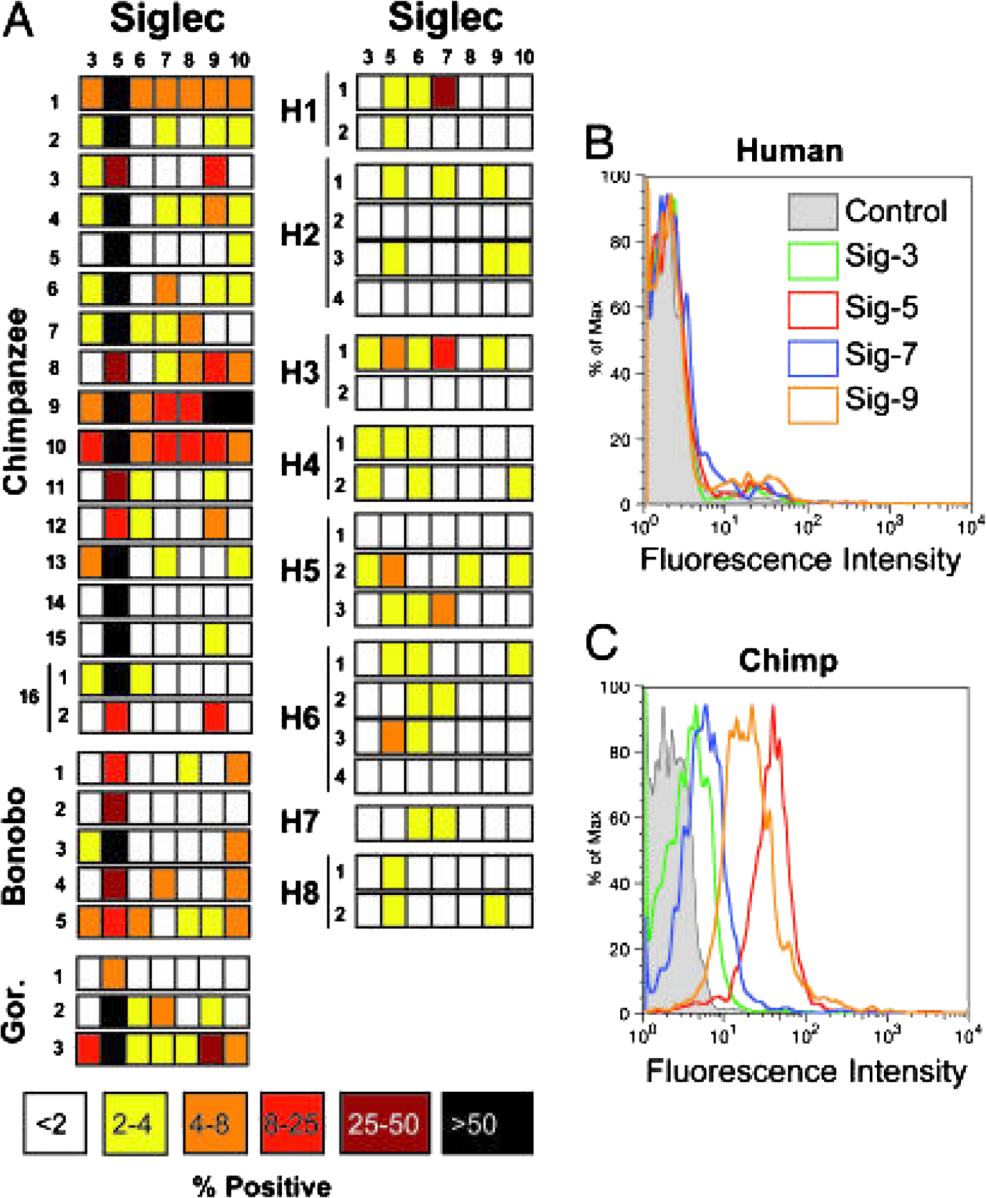
**Variation in sialic acid (Sia)-recognizing Ig-superfamily lectins among primates.** “Expression of CD33rSiglecs on human and great ape lymphocytes. (**A**) Percentage of positive lymphocytes for each Siglec Ab (staining above negative controls) for 16 chimpanzees, 5 bonobos, and 3 gorillas are shown, as well as data for 8 humans (the latter were tested on one or more occasions). Examples of flow cytometry histograms of human (**B**) and chimpanzee (**C**) lymphocytes using Abs recognizing Siglec-3, Siglec-5, Siglec-7, and Siglec-9 (y axis: normalized cell numbers expressed as percent of maximum cell number detected). In later samples examined, low levels of Siglec-11 staining (<5% positive) were occasionally detected on lymphocytes in both great apes and humans (data not shown)” [156].

It is well known that humans and nonhuman primates respond differently to infections. For example, untreated humans usually progress to AIDS when infected with HIV, are susceptible to malaria (except those with sickle cell anemia), have different reactions to hepatitis B and C than nonhuman primates and, appear more susceptible to many cancers and Alzheimer’s disease [153-155]. Barreiro et al. [154] studied gene expression levels in monocytes from humans, chimpanzees, and rhesus macaques and found that all three species demonstrated “the universal Toll-like receptor response” when stimulated with lipopolysaccharide (LPS). However they also discovered that only 58% of genes identified in the Toll-like receptor response “showed a conserved regulatory response to stimulation with LPS,” and only 31% of those genes demonstrated the same conserved regulatory response when exposed to viruses or bacteria. Barreiro et al. also discovered that 335 genes in humans are unique among the species in responding to LPS, with 273 genes responding only in chimpanzees, and 393 only in rhesus macaques [154]. Even in conserved processes, there are going to be significant differences that influence the outcomes from disease perturbations. Significant differences in the details of conserved processes (also illustrated by Figure 1[156]) mean that there are differences in the initial conditions of the complex system and this has major implications for inter-species extrapolation.

The implications of the various properties of complex systems also become apparent when scientists study processes such as preimplantation embryonic development (PED). PED is thought to be highly conserved among species which led Xie et al. [157] to study gene expression profiles in embryos from humans, mice, and cows. They found that: “40.2% orthologous gene triplets exhibited different expression patterns among these species.” Differences in expression profiles have implications for drug and disease response.

The *Cdc14* gene was discovered in the yeast *Saccharomyces cerevisiae* and is classified as a dual-specificity phosphatase. It has since been found in many organisms including humans. Human Cdc14B fulfills the role, in yeast, of the yeast gene *Cdc14*. Because the yeast gene plays a role in regulating late mitosis, it was assumed the gene would have the same role in mammals. In actuality, neither *Cdc14A* nor *Cdc14B* are necessary for cell-cycle progression in humans [158]. Thus, we have a conserved gene but not a conserved function.

Pyrin proteins have been found to be ubiquitous in mammals. Pyrin-only protein 2 (POP2) was found in humans and thought to be important in inflammatory diseases. Atianand et al. [159] studied mice but did not find POP2. They then discovered that POP2 was not in rodents or many other mammals but was present in chimpanzees (*Pan troglodytes*) and rhesus macaques (*Macaca mulatta).* Moreover, the chimpanzee POP2 was identical to humans POP2 at both the DNA and protein levels but the macaque POP2 was not.

Conserved processes act, are affected by, or interact at multiple levels of organization. As Cairns-Smith points out, proteins, catalysts, nucleic acids, membranes, and lipids are interlocked and all are dependent on the others for their production. Cairns-Smith summarizes by stating: “Subsystems are highly interlocked . . . The inter-locking is tight and critical. At the centre everything depends on everything” [[81] p39]. The same perturbation may result in different effects or outcomes for different levels of organization in the same intact system. This further complicates our ability to predict outcomes between two intact living complex systems. Thus it appears that a perturbation of complex system S_1_ containing conserved process P_1_ resulting in outcome O_1_ will not necessarily result in O_1_ in the very similar complex system S_2_ that also has P_1_.

We will now examine in more detail the response of organisms to inhalational anesthetics and anti-neoplastic agents in order to illustrate what can and cannot be extrapolated between species knowing that species are acted on and affected by the fundamental principles of geometry, chemistry, and physics as well as shared conserved processes.

### Conserved processes in anesthesia

General anesthesia by means of inhalational anesthetics (IAs) provides us with an excellent opportunity to examine where the effects of conserved processes can and cannot be extrapolated between species. We expect to see various effects at different levels of organization and in different modules. We also anticipate effects on emergent properties. Because IAs act on the system as a whole, we expect to see effects that cannot be predicted from reductionism. This has implications for what can be expected in terms of predicting human response by studying a different species or perhaps even a different individual. Therefore, both the primary effect of the anesthetic agent as well as the side effects may vary.

Broadly speaking, general anesthesia in humans and animals is defined by amnesia, controlled insensitivity and consciousness, and immobility. It has been observed that most, if not all, extant vertebrate species exhibit an anesthetic-like response to a wide variety of chemicals that seemingly have little in common. This has been termed the *universal response*. Multiple mechanisms for the universal response have been postulated and this is an area of intense current research [160-164]. There seems to be general agreement that ligand gated ion channel (LGIC) protein receptors are involved as well as possible effects on the cellular membrane. Regardless of the exact details, the conservation of mechanisms can be seen in that inhalational anesthetics (IAs) have observable effects on motor or motility responses in vertebrates and invertebrates [165-168], tactile plants [169] and ciliated protists [170]. (We note that this is probably an example of an exaptation, specifically a spandrel, rather than an adaptation [171-173].) Interestingly, effects have even been observed in *S. cerevisiae* (Baker’s yeast) [174], suggesting that crucial aspects of the universal response go beyond metazoans to include Eukaryotes. Moreover, IAs have been shown to have effects on membrane composition in prokaryote species [163,175] e.g., *A. laidlawii*[176,177], *Bacillus halodurans*[175] and *E. coli*[178] and the single-celled eukaryote tetrahymena [179,180] (a ciliated protozoan). The universal response appears to date far back in evolutionary time and strongly suggests that the mechanism has been conserved among species.

However, there are differences in outcomes with respect to IAs. Humphrey et al. [181] studied genes in *Caenorhabditis elegans* and *Drosophila melanogaster* in order to assess the function of genes thought involved in the response to IAs. They found that a gene in *C elegans*, *unc-79*, and a gene in *Drosophila*, *narrow abdomen (na)*, were related to each other and play a conserved role in response to anesthetics. However, mutations in each gene produced unique changes in sensitivity to IAs. The sensitivity to halothane, an IA, was increased but the sensitivity to enflurane, a different IA, was unchanged or perhaps even lowered. This is perplexing because one would have expected the two inhalational agents to be affected in a similar fashion by the mutation. The gene *unc-79* appears to be a post-transcriptional regulator of *na*, thus the genes operate in the same pathway. Interestingly, both genes are also associated with similar phenotypes regarding locomotion: “fainting” in *C. elegans* and “hesitant walking” in *Drosophila*.

Stimulation of the conserved processes controlling the universal response results in clinically significant variability among humans, even though the minimum alveolar concentration (MAC) for IAs for most species is approximately the same. MAC is the most often used metric to assess IA potency. However, the concept of MAC implies variability. MAC_50_, simply called MAC in anesthesiology, is the minimum alveolar concentration necessary to suppress movement in response to painful stimuli in 50% of subjects [182]. MAC is significantly variable among humans depending on a number of factors including age and sex. Why is this the case?

Sonner et al. reported, “one hundred forty-six statistically significant differences among the 15 strains [of mice] were found for the three inhaled anesthetics (isoflurane, desflurane, and halothane)” [164]. They concluded that multiple genes must be involved in anesthetic potency. Wang et al. developed two strains of mice that manifested different sensitivities to isoflurane [183]. MAC is an example of a phenomenon controlled by quantitative trait loci [184], which may explain in part why, while one can obtain a rough approximation of MAC by studying other species, there will still be clinically significant differences.

IAs also function at different levels of organization and on modules in addition to the one involved in the universal response. The side effects of the same chemical that produce an effect on the conserved receptors or other processes vary greatly from species to species and in some cases, even from person to person. A good example is the case of isoflurane and coronary steal. In the 1980s, there was heated controversy regarding the administration of the inhalation anesthetic isoflurane to patients with heart disease. The controversy centered on research using canines that indicated that the drug caused myocardial ischemia during certain situations in patients with coronary disease. The phenomenon appeared to result from isoflurane causing dilation of the normal coronary arteries, and thus blood being shunted away from the occluded coronary arteries; the arteries and tissues that most needed it. This was called *coronary steal*. Further, this situation was worsened by a decrease in blood pressure; a condition that often occurs during general anesthesia with IAs. This supposed danger, based almost entirely on studies in canines, was seized on by many in the anesthesiology community as dogma [185,186].

This was an interesting reaction from clinicians for two reasons. First, experiments with other species had failed to demonstrate coronary steal [187,188] and second, anesthesiologists had not noticed ischemic changes associated with isoflurane despite much use of the agent. The situation was also troublesome because isoflurane was a needed addition to an anesthesiologist’s armamentarium when initially approved for clinical practice. Six years after its introduction, it was the most frequently used IA, in part because of the favorable properties of the drug [185]. Further studies continued to demonstrate varying effects intra- and inter-species [189,190]. Ultimately, studies began to appear that suggested isoflurane was in fact cardio-protective. The mechanism for this protection was called “preconditioning and involves the opening of adenosine triphosphate-dependent potassium channels” [191]. Isoflurane went from being contraindicated in patients with coronary artery disease to being the drug of choice in such patients. Studies from animals, specifically dogs, figured heavily in forming both, mutually exclusive, conclusions.

Just as with the homeobox, miRNAs, and the response to inflammation, there are differences among species in how the conserved process known as the universal response to anesthesia manifests. Clinically, these differences are significant and limit the amount of information that can be extrapolated between species even when the underlying process is conserved. Inhalation anesthetics are also a good example of why, when the level of organization or module being examined changes, extrapolation breaks down. The same chemical that induces general anesthesia in a dog will probably result in the same effect in humans but the dose may vary in a clinically significant fashion and the side effects will most likely vary, as the conserved process does not dictate the side effects. Differences in outcomes from perturbations like the ones we have seen above have been explained by evolution-based species-specific differences, for example background genes, mutations, expression levels, and modifier genes [192-209].

### Anti-neoplastic drugs acting on mitosis

As discussed, a relationship exists between BSA and many physiological parameters [210]. For example, Reagan-Shaw, Nihal, and Ahmad state: “BSA correlates well across several mammalian species with several parameters of biology, including oxygen utilization, caloric expenditure, basal metabolism, blood volume, circulating plasma proteins, and renal function” [211]. Dosing algorithms for first-in-man (FIM) trials are based on the assumption that there is a one-to-one dose scale between humans and animals when BSA is taken into account [212]. The first study suggesting a relationship between dose and body surface area was performed by Pinkel in 1958 [210] involving anti-neoplastic agents, drugs where the effects and side effects are largely the same—cell death. Subsequently, Freireich et al., [213] studied 18 anti-neoplastic drugs in six animal species and concluded that the maximum tolerated dose (MTD) for humans was 1/12 of the dose in mice that resulted in the death of 10% of the mice (LD10). They also noted that the MTD was 1/7 of the LD10 in rats. These were also the ratios for converting from a mg/kg dose to a dose based on BSA. Fifty anti-neoplastic drugs were then studied using this formula and all were reportedly introduced into human trials without incident [214,215]. The standard for FIM doses then became the 1/10^th^ the LD10 for mice. Actually Freireich recommended a starting dose of 1/3^rd^ the LD10 not 1/10^th^ but that changed over time. The 1/3^rd^ recommendation was found to be too large for FIM and was changed to 1/10^th^[216]. More studies appeared to confirm the 1/10^th^ value [217].

The above makes a *prima facie* case that animal models can predict a starting dose for humans in clinical trials for anti-neoplastics. Further substantiating this is the fact that anti-neoplastics are not always metabolized by the liver [218], thus possibly eliminating a complex system from consideration. Cell division by mitosis is arguably the most conserved process one can find in biology and the traditional drugs for treating cancer act by interfering with mitosis. (Newer drugs act on targeted pathways as opposed to the cell cycle.) Anti-neoplastics kill the cells that are dividing most rapidly—the cancer cells. However, hair cells, cells in the bone marrow, and cells in the gut also divide at a similar rate such that anti-neoplastics can affect them. Thus, in part, the effects and side effects of anti-neoplastics are the same—cell death. The problem with traditional anti-neoplastics is that they do not discriminate adequately.

Anti-neoplastic drugs are unique in medicine in that: 1) they are nonspecific; 2) long term toxicities are anticipated and accepted because the patient frequently does not have any other viable options; 3) the effects and side effects of the drugs are the same—cell death; and 4) they act on a universally conserved process—mitosis. This is why body surface area appears to be so effective for calculating FIM dose. Whereas, when one is examining effects and side effects of drugs based on interactions at the level of organization where complexity is relevant, for example metabolism [219-229], there are simply too many other factors to allow for the expectation of one-to-one correlations. Species-specific differences create perturbations in the complex system thus the differences among species outweigh the similarities [13-16,18,21-41].

However, in the final analysis even the FIM dose of the anti-neoplastic agents cannot be reliably ascertained based on BSA. Most anti-neoplastics are effective only at doses near the maximum tolerated dose and the drugs are given in an escalating fashion during clinical trials. “Patients treated at the lower end of the dose escalation strategy are unlikely to receive even a potentially therapeutic dose since most cytotoxic drugs are only active at or near the MTD” [217]. Differences among species in dose response for anti-neoplastics are due in part to differences in pharmacokinetics [217,230-232], which cannot be accounted for based on BSA. Brennan et al. state that: “While proper determination of drug doses can be complicated within the same species, it can be an incredible challenge and burden between species” [233]. Brennan et al. continue by pointing out that metabolism and clearance differ among species and that “…the liver, kidneys and hematopoietic system between species may have significant differences in their sensitivity to chemotherapeutic agents. None of these factors are taken into account with the use of the species-specific dose calculations” [233]. They recommend area under the curve (AUC) for calculating FIM dose but then concede: “However, there are numerous examples in which the species-specific conversion dose varies significantly from the AUC guided dose and/or far exceeds the animal’s maximum tolerated dose.” They then list examples from pediatrics where the recommended and actual doses differ significantly [233].

Horstmann et al. [234] reviewed 460 Phase I National Cancer Institute trials involving 11,935 adults that occurred between 1991 and 2002. Approximately 25% of the trials were FIM trials. Horstmann et al. found that serious nonfatal effects occurred in 15% of the patients undergoing single chemotherapy, with 58 deaths that were probably treatment-related [234,235]. Concern has also been expressed that animal models have derailed anti-neoplastics that would have been successful in humans [30,235-239].

FIM dose based on animal models is ineffective for predicting dose for other drug classes as well-TGN1412 being a recent notable example [26,240,241]. An unnamed clinician, speaking of toxicity trials for new drugs in general in humans, was quoted in *Science,* stating, “If you were to look in [a big company’s] files for testing small-molecule drugs you’d find hundreds of deaths” [242]. Chapman reinforced this stating: “. . . but other incidents of harm [besides TGN1412], even death, to participants in Phase I trials, some then known and other unpublicized, had taken place” [235]. It is also important to note that the 1/3^rd^ or 1/10^th^ safety factor is fabricated. Perlstein et al. state: “Due to uncertainty in translating animal model findings to humans, particularly for unprecedented mechanisms, a wide dose range (1000-fold) is expected to cover the entire exposure–response curve” [243]. Extrapolating from species to species should not require *fudge factors* if the process is truly science-based. In Phase I trials, where FIM or first in human (FIH) occurs, scientists want to characterize the drug’s PK properties and safety margins [244]. Wexler and Bertelsen summarize the situation when they state: “Although allometric scaling techniques continue to provide poor predictive estimates for human pharmacokinetic parameters, FIH starting doses are selected with substantial safety factors applied to human equivalent dose, often in excess of regulatory guidelines. . . . Approaches that could enhance the predictive nature of a compound’s disposition and adaptive nature of FIH studies could provide a tremendous benefit for drug development” [245]. FIM for all classes of drug could be easily accomplished using microdosing [246-248] with the first dose of one nanogram [249,250] and increasing subsequent doses to the desired endpoint.

Finally, one must recall that 95% [31,251,252] of anti-neoplastic agents fail in clinical trials. Oncology drugs fail more frequently in clinical trials than most other categories [253,254] and a higher percentage of anti-neoplastic drugs fail in Phase III trials than drugs from any other category [255]. Reasons for the attrition include the fact that most of the effects and side effects, even of the anti-neoplastic agents, when placed into the context of a complex system, are not predicted from animal studies. Interfering in mitosis is a universal phenomenon but the degree and success of that interference varies. The FIM dose estimation is apparently successful because the level of organization in question is very basic and conserved and because the dose is lowered even further by fudge factors. Picking a starting dose based on the most toxic substances in nature [249,250] would be more scientific. The apparent success also breaks down because the types of cancers in humans differ from those in animals, the genetic background of humans varies from that in animals, and because the reality of a complex system—the interactions of all the other systems (for example how the drugs are eventually metabolized and eliminated and how those metabolites interact with other systems and so on)—eventually appear. These are the problems that cannot be solved by animal models and are why the attrition rate is 95%. Weinberg stated: “it’s been well known for more than a decade, maybe two decades, that many of these preclinical human cancer models have very little predictive power in terms of how actual human beings—actual human tumors inside patients—will respond . . . preclinical models of human cancer, in large part, stink . . . hundreds of millions of dollars are being wasted every year by drug companies using these [animal] models” [236]. Others have also pointed out the inadequacy of animal models of cancer, including genetically modified animal models [41,214,252,256-261].

### Conserved processes in light of systems biology

As the level of organization in a complex system increases, we expect to see an increase in the number of emergent properties as well as more overall interactions. A gene or process that has been conserved will interact with the intact whole organism yielding new processes and states. Perturbations of these conserved genes or processes will thus likely result in new states not seen in other organisms that share the conserved processes; perhaps not even in organisms of the same lineage (clade) or species.

The lack of appreciation for the differences between levels of organization and other properties of complex systems is apparent in the following from Kardong [[262] p2], writing in his textbook of comparative vertebrate anatomy: “For example, by testing a few vertebrate muscles, we may demonstrate that they produce a force of 15 N (newtons) per square centimeter of muscle fiber cross section. Rather than testing all vertebrate muscles, a time-consuming process, we usually assume that other muscles of similar cross section produce a similar force (other things being equal). The discovery of force production in some muscles is extrapolated to others. In medicine, the comparative effects of drugs on rabbits or mice are extrapolated to tentative use in humans.” At the level of organization where one studies the force generated by muscle fibers, no doubt inter-species extrapolation is useful, but that is an entirely different level from where drug actions occur.

Indeed the successes from using animal models have been examples of perturbations occurring at subsystems that can be described as simple systems and or outcomes or characteristics that apply on the gross level of examination. For example, the Germ Theory of Disease applies to humans and animals. The immune system reacts to foreign entities in a manner that is grossly similar across species lines. The details of immunity are clinically very different, for example HIV infection leads to AIDS in humans but not chimpanzees [263-265]. Nevertheless, grossly, inflammation, white blood cells, and antibodies are identifying characteristics of the immune system in the phylum Chordata. Likewise, while the heart functions to circulate the blood in mammals, the diseases various mammalian hearts are subject to differ considerably [266-272]. The failures of animal models have occurred when attempting to extrapolate data from higher levels of organization, levels where complexity is an important component in the system or subsystem under consideration. For example, a drug that has passed animal tests and is in Phase I human clinical trials has only an 8% chance of making it to market [273]. Over 1,000 drugs have been shown to improve outcomes in cerebral ischemia in animal models but none, save aspirin and thrombolysis, which were not animal-based discoveries, have been successful in humans [35,274-277]. The animal model for polio, monkeys, revealed a pathophysiology that was very different from that of humans [278-281]. Extracranial-intracranial bypass for inoperable carotid artery disease was successful in animals but results in net harm for humans [282-285].

**Figure 2:**
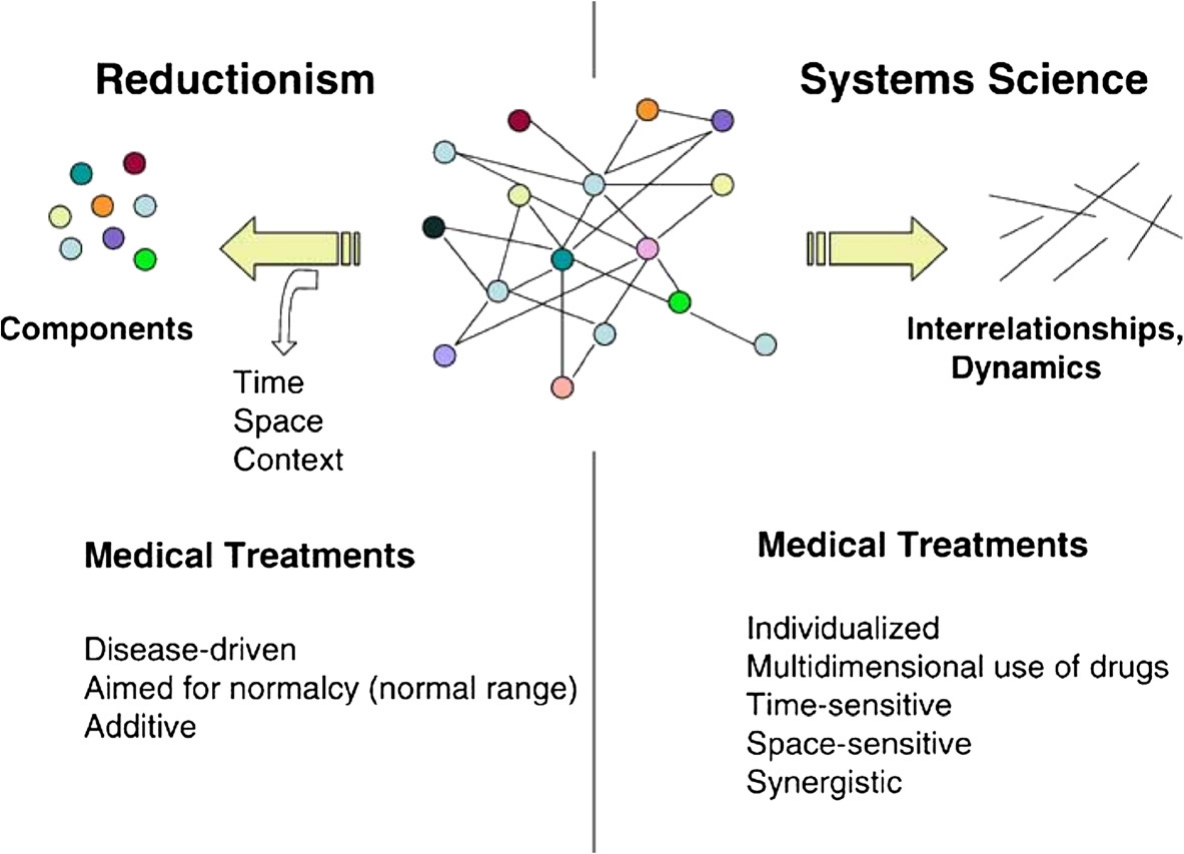
Reductionism versus systems biology.

Most diseases are multifactorial hence it should come as no surprise that conserved processes play a small, although at times important role, in major diseases like heart disease, cancer and stroke. The field of systems biology was formed in part in an attempt to place the parts of molecular biology and genetics in the larger context of the human system; the system that actually responds to drugs and disease. An editorial in *Nature* asks: “What is the difference between a live cat and a dead one? One scientific answer is 'systems biology'. A dead cat is a collection of its component parts. A live cat is the emergent behaviour of the system incorporating those parts” [286]. According to the Department of Systems Biology at Harvard Medical School: “Systems biology is the study of systems of biological components, which may be molecules, cells, organisms or entire species. Living systems are dynamic and complex, and their behavior may be hard to predict from the properties of individual parts” [287]. Systems biology [288] takes a top-down approach as opposed to reductionism, which evaluates organisms from the bottom-up. Systems biology is concerned more with networks than individual components, although both are studied. It also recognizes the importance of emergent phenomena. (See Figure 2[79]). Such top-down approaches are used by the fields commonly referred to as “Omics,” for example: interactomics, metabolomics, proteomics, transcriptomics, and even fractalomics [289].

Nobel laureate Sydney Brenner, in 1998, emphasized that the interactions of components was important in understanding an organism [290]. Only by studying proteins and processes in the context of their systems can we expect to understand what happens to the intact organisms as a result of these processes and genes. Further, evolution uses old pathways and processes in different ways to create novelty [1,133]. Everything is context dependent. Noble stresses that in order to predict how drugs will act, one must understand “how a protein behaves in context” at higher levels of organization [291].

Heng [292], writing in *JAMA* states that, because of reductionism, biological scientists have sought individual components in a disease process so they could intervene. A linear cause and effect relationship was assumed to exist. Heng cites diabetes intervention in an attempt to control blood glucose and cancer therapies as examples. He points out that while this has worked well in many cases, very tight control of blood glucose was recently found to increase the risk of death [293]. Along the same lines, chemotherapies for cancer have decreased the size of the tumors but at the expense of an increase in frequency of secondary tumors and a very adversely affected lifestyle. Furthermore, most chemotherapy does not prolong life or result in a longer, high quality life [294-296]. Instead of focusing on small modules or components of a system, complexity theory mandates that biomedical science look at the system as a whole.

Closely related to systems biology are the concepts of personalized medicine and pharmacogenomics [226,297-305]. It has long been appreciated that humans respond differently to drugs and have different susceptibilities to disease. Based on studies of twins, there appears to be a genetic component to susceptibility to leprosy, poliomyelitis and hepatitis B, as well as response to opioids [306-309]. Other infectious diseases that appear to have a genetic component to susceptibility include HIV, Hepatitis C, malaria, dengue, meningococcal disease, variant Creutzfeldt–Jakob disease and perhaps tuberculosis among others [310]. Differences in drug and disease response are manifest among ethnic groups [311-319] and sexes [320-326]. Even monozygotic twins manifest differences in response to such perturbations [107,108,113-116]. Rashmi R Shah, previous Senior Clinical Assessor, Medicines and Healthcare products Regulatory Agency, London stated in 2005: “During the clinical use of a drug at present, a prescribing physician has no means of predicting the response of an individual patient to a given drug. Invariably, some patients fail to respond beneficially as expected whereas others experience adverse drug reactions (ADRs)” [327].

Similarly, Allen Roses, then-worldwide vice-president of genetics at GlaxoSmithKline (GSK), said fewer than half of the patients prescribed some of the most expensive drugs derived any benefit from them: “The vast majority of drugs - more than 90% - only work in 30 or 50% of the people.” Most drugs had an efficacy rate of 50% or lower [328]. Because of differences in genes, like SNPs, all children may not currently be protected by the same vaccine [329,330]. It is estimated that “between 5 and 20 per cent of people vaccinated against hepatitis B, and between 2 and 10 per cent of those vaccinated against measles, will not be protected if they ever encounter these viruses” [330]. In the future such children may be able to receive a personalized shot. Currently, numerous drugs have been linked to genetic mutations and alleles. See Table 5[303] and Table 6[331]. The number of personalized medicine products has increased from 13 in 2006 to 72 as of 2012 [332].

**Table 5:** Examples of drugs with genetic information in thier labels

**Drug**	**Sponsor**	**Indication**	**Gene or genotype**	**Effect of genotype**	**Clinical directive on label**
Abacavir (Ziagen)	GlaxoSmithKline	HIV-1	HLA-B*5701	Hypersensitivity	Black-box warning. "Prior to initiating therapy with abacavir, screening for the HLA-B*5701 allele is recommended." "Your doctor can determine with a blood test if you have this gene variation."
Azathioprine (Imuran)	Prometheus	Renal allograft transplantation, rheumatoid	TPT*2TPT*3Aand TPMT*3C	Severe myeloxicity	"*TPT* genotyping or phenotyping can help identify patients who are at an increased risk for developing Imuran toxicity." "Phenotyping and genotyping methods are commercially available."
Carbamazepine (Tegretol)	Novartis	Epilepsy, trigeminal neuralgia	HLA-B*1502	Stevens-Johnson syndrome or toxic epidermal necrolysis	Black-box warning: "Patients with ancestry in genetically at-risk populations should be screened for the presence of HLA-B*1502 prior to initiating treatment with Tegretol. Patients testing positive for the allele should not be treated with Tegretol." "For genetically at-risk patients, high-resolution HLA-B*1502 typing is recommended."
Cetuximab (Erbitux)	Imclone	Metastatic colorectal cancer	*KRAS* mutations	Efficacy	"Retrospective subset analyses of metastatic or advanced colerectal cancer trials have not shown a treatment benefit for Erbitux in patients whose tumors had KRAS mutations in codon 12 or 13. Use of Erbitux is not recommended for the treatment of colorectal cancer with mutations."
Clopidogrel (Plavix)	Bristol-Myer Squibb	Anticoagulation	CYP2C19*2*3	Efficacy	"Tests are available to identify a patient’s CYP2C19 genotype; these tests can be used as an aid in determining therapeutic strategy. Consider alternative treatment or treatment strategies in patienrs identified as CYP2C19 poor metabolizer."
Irinotecan (Camptosar)	Pfizer	Metastatic colorectal cancer	UGT1A1*28	Diarrhea neutropenia	"A reduction in the starting dose by at least one level of Camptosar should be consider for patients knows to be homozygous for the UGT1A1*28 allele. "A laboratory test is available to determine the UGT1A1 status of patients."
Pantumumab (Vectibix)	Amgen	Metastatic colorectal cancer	*KRAS* mutations	Efficacy	"Retrospective subset analyses of metastatic colorectal cancer trials have not shown a treatment benefit for Vectibix in patients whose tumors had KRAS mutations in codon 12 or 13. Use of Vectibix is not recommended for the treatment of colorectal cancer with these mutations."
Transtuzumab (Herceptin)	Genetech	HER2-positive breastcancer	HER2 expression	Efficacy	"Detection of HER2 protein overexpression is necessary for selection of patients appropriate for Herceptin therapy because these are the only patients studied and for whom benefit has shown." "Several FDA-approved commercial assays are available to aid in the selection of breast cancer and metastatic cancer patients for Herptin therapy."
Wafarin (Coumadin)	Bristol-Myer Squibb	Venous thrombosis	CYP2C9*2*3 and VKORC1 variants	Bleeding complications	Includes the following table: Range of Expected Therapeutic Warfarin Doses Based on CYP2CP and VKORC1 Genotypes.

*All drug labels were accessed through Drugs @FDA at www.accessdata.fda.gov/scripts/cder/drugsatfda. HIV-1 denotes human immunodeficiency virus type 1, *TPMT* thiopurine methyltransferase, *UGT1A1* UDP glucuronosyltransferanse 1 family polypeptide A1, and *VKORC1* vitamins K epoxide reductase complex subunit 1

**Table 6:** The most significant genetic predictors of drug response

**Organ or system involved**	**Associated gene/allele**	**Drug/drug response phenotype**
**Blood**		
Red blood cells	*G6PD*	Primaquine and others
Neutrophils	TMPT*2	Azathioprine/6MP-induced neutropenia
	UGT1A1*28	Irintotecan-induced neutropenia
Plates	CYP2C19*2	Stent thrombusis
Coagulation	CY2C9*2, *3, VKORC1	Warfarin dose-requirement
Brain and peripheral nervous system		
CNS depression	CYP2D6*N	Codeine-related sedation and respiratory depression
Anaesthesia	*Butyrylcholinesterase*	Prolonged apnoea
Peripheral nerves	*NAT-2*	Isoniazid-induced peripheral neuropathy
Drug hypersesitivity	HLA-B*5701	Abacavir hypersensitivity
	HLA-B*1502	Carbamazepine-induced Steve Johnson syndrome (in some Asian groups )
	HLA-A*3101	Carbamazepine-induced hypersensitivity in Causians and Japanese
	HLA-B*5801	Allopurinol-induced serious cutaneous reactions
Drug-induced liver injury	HLA-B*5701	Flucloxacillin
	HLA-DR81*1501-DQ81*0602	Co-amoxiclav
	HLA-DR81*1501-DQ81*0602	Lumiracoxib
	HLA-BR81*07-DOA1*02	Ximelagatran
	HLA-DQA1*0201	Lapatinib
**Infection**		
HIV-1 infection	*CCRS*	Maraviroc efficacy
Hepatitis C infection	*IL288*	Interferon-alpha efficacy
**Malignancy**		
Breast cancer	*CYP2D^*	Response to tamoxifen
Chronic myeloid leukaemia	*BCR-ABL*	Imatinib and other tyrosine kinase inhibitors
Colon cancer	*KRAS*	Cetuximab efficacy
GI stromal tumours	*c-kit*	Imatinib efficacy
Lung cancer	*EGFR*	Gefinib efficacy
	*EML4-ALK*	Crizotinib efficacy
Malignant melanoma	*BRAF V600E*	Vemurafenib efficacy

When animals were being used as models in the 19^th^ century, many of the scientists who were using them had not accepted evolution and believed that animal parts were interchangeable with their human counterparts [60,62,63]. Given that we now understand that intra-human variation results in such markedly different responses to drugs and disease, attempting to predict human response from animal models, even for perturbations acting on conserved processes, seems unwarranted. Yet, despite the implications of personalized medicine [22], some scientists continue to commit the fallacy described by Burggren and Bemis: “Yet the use of ‘cockroach as insect,’ ‘frog as amphibian,’ or ‘the turtle as reptile’ persists, in spite of clear evidence of the dangers of this approach. Not surprisingly, this type of comparative physiology has neither contributed much to evolutionary theories nor drawn upon them to formulate and test hypotheses in evolutionary physiology” [[333] p206]. Comparative research will yield a nice comparison of the trait or process among species or phyla. However, one simply cannot assume that the outcome from a specific perturbation in, say the cockroach, will be seen in insects in general and this concept becomes even more important when relying on animal models for medical interventions in humans.

## Conclusion

A perturbation of living complex system S_1_ containing conserved process P_1_ resulting in outcome O_1_ will not result in O_1_ in the very similar living complex system S_2_ that also has P_1_ often enough to qualify S_1_ as a predictive modality for S_2_ when the trait or response being studied is located at higher levels of organization, is in a different module, or is influenced by other modules. However, when the examination of the conserved process occurs at the same or lower level of organization or in the same module, and hence is subject to study solely by reductionism, then extrapolation is possible. We believe this is a valuable principle.

Our current understanding of evo devo, evolution in general, complexity science, and genetics allows us to generalize regarding trans-species extrapolation, even when conserved processes are involved. Shanks and Greek:

"Living complex systems belonging to different species, largely as a result of the operation of evolutionary mechanisms over long periods of time, manifest different responses to the same stimuli due to: (1) differences with respect to genes present; (2) differences with respect to mutations in the same gene (where one species has an ortholog of a gene found in another); (3) differences with respect to proteins and protein activity; (4) differences with respect to gene regulation; (5) differences in gene expression; (6) differences in protein-protein interactions; (7) differences in genetic networks; (8) differences with respect to organismal organization (humans and rats may be intact systems, but may be differently intact); (9) differences in environmental exposures; and last but not least; (10) differences with respect to evolutionary histories. These are some of the important reasons why members of one species often respond differently to drugs and toxins, and experience different diseases. Immense empirical evidence supports this position ([14] p358)."

The failures of animal models as a predictive modality for human response to disease and drugs, even when such perturbations are acting on conserved processes, can be explained in the context of evolved complex systems. One does not need to study every such perturbation in every species in order to conclude that the animal model will not be a predictive modality for humans when perturbations occur at higher levels of organization or involve different modules or affect the system as a whole. This is not to deny that animal models, as characterized by 3–9 in Table 1, have contributed and will continue to contribute to scientific advancements.

## Competing interests

The authors declare that they have no competing interests.

## Authors' contributions

The authors contributed equally to this paper.

## Authors’ information

Ray Greek, MD has been on faculty in the Department of Anesthesiology at the University of Wisconsin-Madison and at Thomas Jefferson University in Philadelphia. He is currently president of the not-for-profit Americans For Medical Advancement (http://www.AFMA-curedisease.org).

Mark Rice, MD is currently on faculty at the University of Florida (UF). He is chief of the liver transplant division at UF Department of Anesthesiology, has seven US patents, and reviews for several major journals.
